# COVID-19 Pandemic as Risk Factors for Excessive Weight Gain in Pediatrics: The Role of Changes in Nutrition Behavior. A Narrative Review

**DOI:** 10.3390/nu13124255

**Published:** 2021-11-26

**Authors:** Hellas Cena, Lauren Fiechtner, Alessandra Vincenti, Vittoria Carlotta Magenes, Rachele De Giuseppe, Matteo Manuelli, Gian Vincenzo Zuccotti, Valeria Calcaterra

**Affiliations:** 1 Clinical Nutrition and Dietetics Service, Unit of Internal Medicine and Endocrinology, ICS Maugeri IRCCS, 27100 Pavia, Italy; hellas.cena@unipv.it (H.C.); or matteo.manuelli@icsmaugeri.it (M.M.); 2 Laboratory of Dietetics and Clinical Nutrition, Department of Public Health, Experimental and Forensic Medicine, University of Pavia, 27100 Pavia, Italy; alessandra.vincenti01@universitadipavia.it (A.V.); rachele.degiuseppe@unipv.it (R.D.G.); 3 Division of General Academic Pediatrics, Department of Pediatrics, Massachusetts General Hospital for Children, Boston, MA 02114, USA; LFIECHTNER@partners.org; 4 Division of Gastroenterology and Nutrition, Massachusetts General Hospital for Children, Boston, MA 02114, USA; 5 Center for Pediatric Nutrition, Harvard Medical School, Boston, MA 02115, USA; 6 Pediatric Department, “V. Buzzi” Children’s Hospital, 20154 Milan, Italy; vittoria.magenes@unimi.it (V.C.M.); gianvincenzo.zuccotti@unimi.it (G.V.Z.); 7 “L. Sacco” Department of Biomedical and Clinical Science, University of Milan, 20157 Milan, Italy; 8 Pediatric and Adolescent Unit, Department of Internal Medicine, University of Pavia, 27100 Pavia, Italy

**Keywords:** COVID-19, obesity, children, excessive gain, overweight, pediatrics, nutrition, behavior

## Abstract

During the coronavirus disease 2019 (COVID-19) pandemic, social isolation, semi-lockdown, and “stay at home” orders were imposed upon the population in the interest of infection control. This dramatically changes the daily routine of children and adolescents, with a large impact on lifestyle and wellbeing. Children with obesity have been shown to be at a higher risk of negative lifestyle changes and weight gain during lockdown. Obesity and COVID-19 negatively affect children and adolescents’ wellbeing, with adverse effects on psychophysical health, due in large part to food choices, snacking between meals, and comfort eating. Moreover, a markable decrease in physical activity levels and an increase in sedentary behavior is associated with weight gain, especially in children with excessive weight. In addition, obesity is the most common comorbidity in severe cases of COVID-19, suggesting that immune dysregulation, metabolic unbalance, inadequate nutritional status, and dysbiosis are key factors in the complex mechanistic and clinical interplay between obesity and COVID-19. This narrative review aims to describe the most up-to-date evidence on the clinical characteristics of COVID-19 in children and adolescents, focusing on the role of excessive weight and weight gain in pediatrics. The COVID-19 pandemic has taught us that nutrition education interventions, access to healthy food, as well as family nutrition counselling should be covered by pediatric services to prevent obesity, which worsens disease outcomes related to COVID-19 infection.

## 1. Introduction

At the end of 2019, a novel coronavirus caused a cluster of pneumonia in Wuhan, a city in the Hubei province, in China. It then rapidly spread throughout the world. In February 2020, the World Health Organization (WHO) named the disease induced by this novel virus “COVID-19” (coronavirus disease 2019) and the virus “SARS-CoV-2” (severe acute respiratory syndrome coronavirus 2). The WHO declared COVID-19 a pandemic on 11 March 2020 [1].

Social isolation, semi-lockdown, and “stay at home” orders were imposed upon the population. This was in an effort to reduce public gatherings that would heighten viral exposure and contagion. Many public spaces, such as gyms, swimming pools, theatres, and cinemas, were shut down, resulting in limitation of participation in sports, and physical activity and social interaction and enjoyment [2]. The consequences of those abrupt changes affected the physical and mental health of children and adolescents all over the world, disrupting daily life routine and negatively impacting food choices and eating habits, despite different results reported in the literature [3,4,5,6]. Moreover, the pediatric population with obesity has been shown to be more at risk both for negative lifestyle changes and weight gain during lockdown [6] and worse disease outcomes when infected with COVID-19 [7,8].

This narrative review aims to describe the most up-to-date evidence on the clinical characteristics of COVID-19 in children and adolescents, focusing on the role of excessive weight and weight gain in pediatrics and its adverse effect on psychophysical health, due in large part to unhealthy food choices or access to healthy food and eating behavior including snacking between meals and comfort eating.

## 2. Methods

We proposed a narrative review [9], in an attempt to summarize the literature to answer our research question on the influence of the relationship between obesity and COVID-19 infection on lifestyle in children and adolescents, focusing on eating behavior and food choices. The authors A.V., V.C.M., and R.D.G. independently identified the most relevant published studies in the past 10 years regarding the general effect of obesity and focusing on the last 2 years for the relationship between obesity and COVID-19, in the English literature, including original papers, metanalysis, clinical trials, and reviews. Case reports or series and letters were excluded. Papers published up to August 2021 in each author’s field of expertise were searched with the following keywords (alone or in combination): COVID-19, pandemic, adolescents, children, lifestyle, eating behavior, nutrition, obesity, lockdown, food choice, dietary habits, excessive weight gain, BMI. The following electronic databases were searched: PubMed, Scopus, EMBASE and Web of Science. The contributions were critically reviewed by V.C. and H.C. and collected by V.C., H.C., A.V., V.C.M., and R.D.G. The resulting draft was discussed with all co-authors and the final version was approved by all.

## 3. Epidemiology and Clinical Characteristics of COVID-19 in Childhood and Adolescence

Children of all ages can become ill with coronavirus disease 2019 (COVID-19) [3,10], although it seems that they are affected less than adults [10,11].

Specifically, in the surveillance from various countries updated by the American Academy of Pediatrics, children account for 14.2% of confirmed cases, for 1.3–3.6% of total reported hospitalizations, and for 0.00–0.24% of all COVID-19 deaths [4,11].

In terms of the age distribution of infection, according to data reported between March and December 2020 in the United States, the highest number of cases reported occurred in elementary school children (5–10 years old) [4]. Those data have been confirmed by a recent meta-analysis considering the studies carried out between December 2019 and January 2021 [5]. There is a slight prevalence in males compared to females [5]. As is seen in adults [6], economic and racial/ethnic inequalities exist and racial/ethnic minorities and low-income populations continue to be disproportionately affected by SARS-CoV-2, raising hospitalization and mortality levels [12,13].

The clinical characteristics of COVID-19 infection in children are similar to the manifestations seen in adults; however, children tend to have milder symptoms [14,15,16]. In children, the most common symptoms are mild respiratory ones, including fever, cough, and rhinorrhea [15,17]. In a recent study, the largest systematic review on pediatric COVID-19 [5] published, the percentage of asymptomatic children was 13% and the most represented symptoms were the following: fever, accounting for 63% of cases; followed by cough, 34% of cases [5]; nausea/vomiting, 20% of cases; diarrhea, 20% of cases; and dyspnea, 18% of cases. Other symptoms reported were nasal symptoms, rashes, fatigue, abdominal pain, and neurologic symptoms [5], while cardiovascular symptoms were reported in a minority of cases [5,18].

According to age-group difference in children, dyspnea was shown to be more common in infants, while gastrointestinal symptoms, such as diarrhea and vomiting, as well as headache are more common in the older age groups (>10 years old children) [17].

Although most children show asymptomatic or mild disease and recover promptly, some cases of severe COVID-19 infection, around 3% [3], including fatal cases, have been reported [19,20]. Severe illness in children, as in adults, manifests with dyspnea, central cyanosis, hypoxemia and critical illness with acute respiratory distress, respiratory failure and/or shock [21,22].

A recent systematic review and meta-analysis [23] described that children with previous comorbidities have a higher risk of severe manifestations of COVID-19 and mortality compared to healthy children. Interestingly, it has been shown that childhood obesity (CO) was correlated with a worse COVID-19 prognosis and that CO was the most significant factor associated with the need for mechanical ventilation in children ≥2 years old [24], consistent with what has already been described in adults with obesity affected by severe SARS-CoV-2 infection [25,26]. Behind the factors involved in potentiating severe COVID-19 disease in patients with obesity, a key role is played by inflammation; visceral adiposity has been shown to enhance inflammatory cytokines, such as interleukin-6 and C-reactive protein [27], which have been correlated with increased COVID-19 severe manifestations [28]. Moreover, chronic low-grade systemic inflammation, typical of individuals with obesity, is associated with many co-morbid conditions including atherosclerosis, type 2 diabetes, and hypertension, correlated with more critical outcomes in adults affected by COVID-19 [29]. COVID-19 patients with obesity have suffered more severe lung injury with pulmonary fibrosis due to obesity-induced defective lung mesenchymal stem cells, which may not be able to fight against the viral infection by ineffective tissue repair processes and an ineffective immune response [30].

The main obesity-related risk factors for worse COVID-19 clinical outcomes in adults are present also in children and adolescents [31,32]. Moreover, evidence shows that obesity predisposes to higher COVID-19 mortality even in younger patients, mainly due to enhanced dysregulated inflammatory response, cardiac injury, and increased coagulation activity [33]. Interestingly, the increased prevalence of obesity in children contributes to the shift of the age curve of mortality in countries with higher overweight/obesity rates [34].

Beside obesity, other risk factors for severe COVID-19 in children, as adults, are chronic cardiac disease, respiratory diseases, and diabetes [35,36,37], while, unlike adults age is inversely correlated with severe disease, where children younger than 1 year have the highest risk of severe COVID-19 disease [3,23,35]. Other comorbidities reported in children affected by severe COVID-19 infection are immunological, hematological, and oncological disease with immunosuppression [35,38]. 

Noteworthy, another disorder related to SARS-CoV-2 infection has been described in children. This disorder, initially described as being similar to incomplete Kawasaki disease or toxic shock syndrome [39,40], was later termed Multisystem Inflammatory Syndrome in children (MIS-C) [41,42]. Compared to acute SARS-CoV-2 infection, MIS-C is more severe, requiring critical care support in about 68% of cases [41].

MIS-C was hypothesized to be a post-infectious disease, as many patients affected were SARS-CoV-2 negative [43]. Moreover, the peak of MIS-C has been reported to be around four weeks after SARS-CoV-2 infection [39,41,43]. This lag of time, compatible with the timing of acquired immunity development, supports the post-infectious origin of this syndrome [39,43,44]. The exact mechanism by which SARS-CoV-2 can trigger this exaggerated immune response is still unknown.

There are three slightly different case definitions for MIS-C, according to Centers for Disease Control (CDC), Royal College of Pediatrics and Child Health (RCPCH), and World Health Organization (WHO), as reported in Table 1 [40,45,46,47].

Clinically, MIS-C is usually manifested by persistent fever from three to five days, gastrointestinal symptoms (such as abdominal pain, mimicking appendicitis, vomiting, diarrhea), rash, and conjunctivitis followed by multisystem involvement or shock [41].

In MIS-C, cardiac involvement is a common condition, specifically in terms of myocarditis, pericarditis, and endocarditis [48,49]. Respiratory symptoms are generally due to shock or cardiogenic pulmonary edema, different from severe COVID-19 cases [41,43].

Neurocognitive symptoms have also been reported, such as headache, lethargy, and irritability. Less commonly, more severe conditions may occur, such as encephalopathy, seizures, meningoencephalitis, and/or coma [39,50].

The incidence of MIS-C is uncertain, but it appears to be relatively rare, occurring in less than 1% of children with confirmed SARS-CoV-2 infection [42].

The median age of MIS-C cases was 6 to 12 years [42,43] and comorbidities were reported in a minority of cases, around 20% [41]. The most common comorbidity reported was obesity, present in 7% to 26% of MIS-C cases, depending on case series [50,51]. Another associated risk factor is asthma [48]. Racial/ethnic disparities persist in MIS-C, with black children having higher rates than non-Hispanic white children [12,43].

Laboratory findings in children with documented SARS-CoV-2 are variable. The main laboratory abnormalities show elevated C-reactive protein, serum ferritin, lactate dehydrogenase, D-dimers, procalcitonin, erythrocyte sedimentation rate, serum aminotransferases, and leukocyte count [10,16]. Less common abnormalities include lymphocytopenia, lymphocytosis, and increased creatine kinase myocardial band [10].

In children affected by MIS-C, additional laboratory abnormalities detected are neutrophilia, mild anemia, thrombocytopenia, hypoalbuminemia, hypertriglyceridemia, and increased interleukin-6 (IL-6) levels [24,52]. Laboratory markers of inflammation correlate with severity of illness both in severe COVID-19 cases and in MIS-C cases [24,53].

Interestingly, in patients affected by MIS-C, many endocrinological disturbances are noted, including insulin resistance, glycemic fluctuations, and/or hyperglycemia [54]. This is consistent with findings reporting glucose unbalances in critically ill patients, in which hyperglycemic milieu, aimed at providing fuel for the brain and the immune system in stress conditions [54], is considered a risk factor for adverse outcomes [55]. In order to limit those glycemic metabolic imbalances, regular glucose monitoring may be useful and insulin therapy may be needed [54].

In the literature, interactions between glucose-insulin metabolic disorders and SARS-CoV-2 infection have been reported, both in adults and in children [56,57,58]. Interestingly, those dysmetabolisms have been described both in children affected by type 1 diabetes mellitus [57,58] as well as in those without any glycemic disorder [54]. These glycemic fluctuations are compatible with SARS-CoV-2-induced hyperinflammatory response, which impairs correct functioning of pancreatic β-cells [59].

In adult patients, many comorbidities associated with enhanced inflammation have been reported as risk factors for a more critical condition [30,60,61]. In children, only a few studies have highlighted this issue [39,43,50,51,52], making it hard to draw conclusions. Thus, further research should focus on the identification of the role of proinflammatory comorbidities as a substrate for more severe clinical outcomes even in pediatrics.

## 4. The Effects of COVID-19 on Weight Gain and Obesity

In response to COVID-19, in order to lower virus spread and limit the pressure on the health care system, special measures were implemented by the authorities, such as school closures and home confinement. These restrictions changed children’s and adolescents’ everyday routine, modifying their eating behaviors, available food choices, and physical activity, thus increasing risk of weight gain and obesity development [62].

Different studies, from different parts of the world, showed that, during the lockdown of the COVID-19 pandemic period, children increased their food intake and gained weight [63,64,65,66]. For instance, in China, a general BMI increase in adolescents and young adults was reported with an increased prevalence of obesity in teens (15–17 years) from 10.5% to 12.9% [63]. In Palestine, a study showed a weight gain in 41% of adolescents associated with increased consumption of fried foods, sugary drinks, sweets, and dairy products during home confinement [67,68]. In Poland, changes in dietary intake, such as reduced consumption of fresh fruit, vegetables, and legumes, were associated with weight and BMI increases both in adults and children [65]. In a USA cohort study with 17 million adolescents, the obesity prevalence rose to 15% [67]. Lockdown’s impact on BMI changes in pediatrics showed weight gain, with the highest BMI increases in children already most vulnerable to unhealthy weight gain [66], including children with pre-existing obesity, or black or Hispanic children [66]. In addition, 8-to-12-year-old children had higher rates, perhaps in relation to more screen time [66].

In Italian children, especially in males, an increase in the quantity and a reduction in the quality of meals (with higher intake of potato, meat, and sugary drinks) was described [64]. Such results have also been confirmed by international multicenter studies reporting an increased consumption of comfort food, including sweets and fried food, of up to 20% in adolescents from Spain, Italy, Brazil, Colombia, and Chile during lockdown and an association with higher BMI at younger ages [69]. Weight gain was also correlated with lower socio-economic status, attributable to food insecurity and parents’ concern about financial and health consequences of the viral disease, besides stress and social distancing [68,70].

This becomes more relevant considering that previous epidemics have shown a healthy diet is needed to improve physical and mental health [71].

The widespread weight gain and increased risk of obesity during the “COVID-19 era” in children is related not only to changes in eating behaviors, but also to other factors including physical inactivity [72,73,74], stress [62,70], and increased screen time or TV watching with a consequent increase in snacking [66,75], decreased sleep quality [62,76], and even indoor pollution due to parental smoking during home confinement [62,77].

The measures implemented by the authorities reduced children’s extracurricular and outdoor activities and limited physical activity (PA) [67]. This led to a general decrease in PA and an increase in sedentary behavior [72], factors known to be associated with a reduction in energy expenditure and an increased risk of obesity development [78].

Indeed, PA contributes to daily energy expenditure, lean body mass, and metabolic and psychological profiles [73]. Thus, it helps children to maintain physical and emotional wellbeing and prevent excessive weight gain [73,78,79].

Data collected in the US during the most restrictive period of time (April–May 2020) showed that only around 10% of children practiced team sports through virtual platforms, about 29% participated in physical activity lessons (as martial arts or yoga), and 2.4% participated in online gym programs [67,72]. In addition, adolescents from Latin America, Brazil, and Chile were less active during quarantine and increased their weight [69].

In one study using a microsimulation model to project the impact of the COVID-19 pandemic on childhood obesity in the United States [80], the model predicted that a 2-month school closure alone could result in an increase of the childhood obesity rate by 0.64% in US children of kindergarten age [80].

In addition to the increased risk of obesity development in children with a lean or overweight BMI, special attention should be paid to children already affected by obesity. It is well known that children and adolescents tend to gain more weight during summer vacations than during the school year [81,82,83] as schools provide a structured routine with school meals, physical activity, and a routine that promotes an adequate sleep schedule, three factors implicated in obesity risk [64]. This raise the concern that lockdown could cause unfavorable changes in the lifestyle behaviors of homebound children with obesity [64,84].

Moreover, it was shown that COVID-19 lockdown measures caused increased anxiety in children with severe obesity [85]. This could affect lifestyle [85], with consequent weight gain, consistent with the findings that depression, stress, and anxiety are linked with more severe obesity among youths with obesity [62,86,87].

In addition, the COVID-19 pandemic also caused high levels of parental stress, increasing the difficulty in providing a supportive environment for their children [88]. Parental stress has been associated with an increased risk for childhood obesity in different studies [88,89,90].

## 5. The Effects of Obesity on COVID-19

As discussed above, obesity is the most common comorbidity in severe cases of COVID-19 occurring in children and adolescents [7,24,77,91] and the third most prevalent risk factor among children admitted to ICUs [8]. Particularly noteworthy, the factors involved in worse COVID-19 clinical outcomes include not only obesity itself but also the several comorbidities associated with obesity. [7,8].

Unfortunately, due to fewer pediatric COVID-19 cases compared to adults, there is a paucity of studies available to fully identify risk factors and understand the disease course in these patients. There are a few studies [8,24,91,92,93] published on the negative effects of pediatric obesity on COVID-19 clinical outcomes. In a large cohort of hospitalized children affected by COVID-19 and MIS-C, obesity was found to be an independent risk factor for illness severity and length of hospitalization [91]. Obesity had a more negative impact on children hospitalized with acute COVID-19 compared to those affected by MIS-C [91]. 

Understanding the pathophysiologic interrelationship between pediatric obesity and severe SARS-CoV-2 infection is fundamental to identifying preventive measures and/or clinical interventions [93].

The main factors impairing correct immune system functioning in children with obesity, such as glycemic unbalances, dyslipidemias, proinflammatory state, and respiratory and cardiovascular problems [31,94], have been demonstrated to worsen the clinical outcome in severe acute respiratory syndrome coronavirus 2 infection in adults [92,95,96,97,98,99].

For example, children and adolescents with obesity typically have insulin resistance and hyperinsulinism, causing many health repercussions, in particular on the cardiovascular system [99,100,101]. SARS-CoV-2 was shown to interact with ACE-2 and disrupt pancreatic beta cells’ activity, further aggravating hyperinsulinism and its negative consequences [96].

Moreover, children with obesity can present with dyslipidemias, low levels of HDL-cholesterol, and high levels of LDL-cholesterol, known to cause endothelial dysfunction and consequent atherosclerosis [102,103,104], factors shown to be related to worse COVID-19 clinics [99,105]. In adults with obesity arterial stiffness and atherosclerosis, this have been shown to favor SARS-CoV-2 infection of the endothelium and to be associated with chronic oxidative stress [99].

From the cardiovascular point of view, in addition to the increased risk of atherosclerosis development [103,104] due to metabolic abnormalities, children with obesity may have cardiac anatomical changes, such as left ventricle hypertrophy [106], due to hypertension [106], leading to endothelial injuries [103,104] and promoting endothelial attack by SARS-CoV-2 [31,99].

In addition, respiratory physiology may be impaired in children with obesity, mainly due to the pressure exerted by abdominal adiposity on the lungs [107,108], putting them at higher risk of pulmonary infections [101,109], possible complications of COVID-19 [109], and asthma development [107], a known risk factor for MIS-C [48].

Childhood obesity was also shown to alter the immune system by proinflammatory state promotion [110]. In adults with obesity, this immune dysregulation has been related to a worse COVID-19 clinical outcome, ascribable to the intense and deregulated inflammatory reaction, called a cytokine storm, that could be enhanced by the silent chronic hyperinflammatory state typical of both adults and children affected by obesity [99,110,111,112]. Specifically, in youths with obesity, an increase in cytotoxic and effector T cells (Th1 and Th7) and M1-phenotype macrophages coupled with decreased levels of T-reg cells and M2-phenotype macrophages have been reported [112]. Among the molecules involved in this immune derangement, there are adipocytokines, including leptin, cytokines, such as TNF-alpha, IL-6, IL-12, Il-1b, MCP-1, and nitric oxide [112], which have also been correlated with worse COVID-19 clinical outcomes [98,99,113].

Furthermore, the assessment of nutritional status as well as the investigation of nutritional effects on COVID-19 course in the pediatric population [114] have highlighted vitamin D deficiency, a well-documented finding in children with obesity [115], and less commonly, vitamin B12, C, A, E, iron, and folate deficiencies [114].

Another interesting interconnection between obesity and COVID-19 lies in the gut microbiota [116,117,118,119], a complex ecosystem of bacterial species mainly composed of anaerobic microorganisms, belonging to two main phylogenetic lineages: *Firmicutes* and *Bacteroidetes* [116]. In children and adults with obesity, the ratio between the two lineages may be altered, leading to dysbiosis [116,117,118]. In COVID-19 patients, intestinal dysbiosis has been reported [119]. SARS-CoV-2 RNA was found in the feces of infected patients and ACE2 receptors have been shown to also be expressed in the enterocytes of the small intestine [119]. In order to derive conclusions about the role of the microbiome in COVID-19 adults and children, more studies are needed, but these are interesting starting points.

To conclude, it is worth mentioning that obesity has also been linked to worse outcomes in other viral diseases, for instance, the H1N1 epidemic [120] in adults, and the influenza virus in children due to impairment of the cellular immune response and inadequate immunity [31,121].

**Figure 1:**
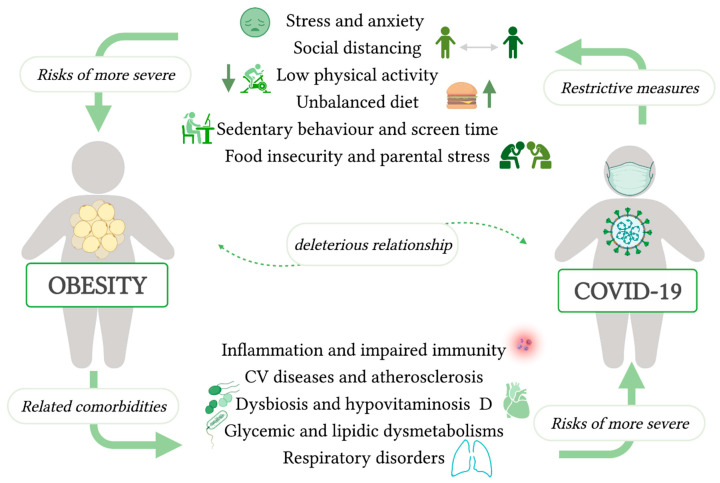
COVID-19/obesity deleterious influences [31]. Created by Biorender.

The deleterious relationship between obesity and COVID-19 is reported in Figure 1. 

## 6. Interactions of Nutrition and COVID-19 Infection

The roles of nutrients in supporting the function of the immune system are well known and it is easy to appreciate that an adequate and balanced supply of micro and macronutrients is essential if an appropriate immune response is to be achieved [84,122]. Although COVID-19 infection cannot be prevented by any specific food or dietary supplements, healthy dietary patterns and optimal nutritional status are key factors for immune function response and support [84,123], especially in pediatrics [31]. 

Since excessive weight gain has a negative impact on nutritional status and immune response [122], it is understandable that overweight and obesity are a main concern for a negative impact on the immune system, increasing the severity and morbidity of lower respiratory tract infections [124] including COVID-19 infection [125]. Indeed, childhood obesity is likely associated with higher COVID-19 susceptibility, severity, and worse prognosis [23,125]. Moreover, obesity-related alterations in pharmacokinetics and pharmacodynamics might reduce the effectiveness of medications [126,127], including antivirals and vaccination [124,128], leaving children with obesity more vulnerable to diseases, mainly explained by chronic systemic sub-clinical inflammation, obesity-related immune system dampening, and decreased cell-mediated immune responses [129].

Moreover, adiposity is associated with higher levels of local and systemic inflammation markers, such as interleukin-6 and C-reactive protein [23,28], which have been positively correlated with COVID-19 susceptibility and severity [28]. 

Evidence shows that a healthy dietary pattern [130] and adequate nutrition positively impact inflammation and immunity, including response to COVID-19 infection [73,122,130,131]. Zhang and Liu, in a systematic review, showed that some nutrients are fundamental for an adequate response against COVID-19 infection, including vitamins A, C, D, and E, omega 3 fatty acids, as well as zinc and iron [132]. In addition, other micronutrients, such as B vitamins (i.e., B_6_, folate, B_12_), selenium, and copper, showed a positive impact on immune function [122,133,134]. Inadequate intake of micronutrients, as well as subclinical deficiencies, may contribute to COVID-19 spread by reducing resistance to infection and easing reinfection [133].

Obesity has been increasingly recognized as a risk factor for several micronutrient deficiencies (MNDs) resulting from: (i) energy-dense and nutrient-poor diets; (ii) increased requirements; and (iii) pharmacokinetics alterations, including distribution, metabolism, and elimination, that could affect micronutrients metabolism [135,136]. Data suggest that some micronutrients, such as vitamin D, may be sequestered in the excessive adipose tissue, leading to less bioavailability [135,137] in children with obesity [138,139,140], aggravated by the absence of outdoor physical activity that stimulates the synthesis of vitamin D [140]. Vitamin D has immunoregulatory properties, regulates the production of chemokines, prevents autoimmune inflammation, and enhances immune cell differentiation [122,137].

Several studies suggested an association between low levels of vitamin D and worse outcomes in COVID-19 patients [141], which makes this issue particularly relevant in children with obesity where the prevalence of this deficiency is high [138,139]. It has been hypothesized that vitamin D deficiency increases infection risk as it seems to have protective effects against respiratory infections and it has been correlated to a higher level of mortality in adult COVID-19 patients [142].

On the other hand, inadequate intake of vitamins A, E, and C, due to low consumption of fruits and vegetables in children and adolescents [143], may affect the immune response because of their antioxidant role, which can protect the cell membrane against reactive oxygen species, supporting the protective function of macrophages, neutrophils, and natural killer cells [122,144]. Interestingly, vitamin E supplementation has been shown to increase resistance to infections, including influenza viruses, in animals [145]. Vitamin C supplementation has shown a role in the prevention of respiratory and systemic infection [146]. Vitamin A, beside regenerating mucosal barriers [145], has a key role in adaptive immunity, and the development of T and B cells [145]. Besides, vitamin A deficiency has been shown to impair the Th2 response and promote the immune response to influenza virus infection [31,145].

Furthermore, B vitamins are important cofactors and coenzymes in several metabolic pathways, and it has been reported that they also play important roles in the maintenance of immune homeostasis and regulation [122,147]. Suboptimal plasma levels of these vitamins have been previously reported in pediatrics with a negative impact on nutrition status [148,149,150]. In addition, deficiency of B_6_, folate, and B_12_ may result in hyperhomocysteinemia, which has been associated with several chronic diseases (e.g., cardiovascular disease, neurodegenerative disease) and inflammation [151,152], negatively correlated with COVID-19 outcomes [153].

With regards to iron, children with obesity are at risk for iron deficiency anemia, due to the low nutritional quality and low iron bioavailability of their diet [143], and anemia has been widely demonstrated in this group [154].

Iron could affect several aspects of the host–virus interactions; viral pathogenesis could be influenced by cellular iron status, since viruses co-opt host cellular processes to replicate using iron-dependent proteins [155]. Iron deficiency might protect against certain microbial infections including malaria [156] and iron supplementation could exacerbate malaria risk in children in endemic areas in the absence of control measures [157,158]. Moreover, excess iron increases siderophilic bacterial infection risk [159] and gastrointestinal and respiratory infections have been reported in trials of childhood iron supplementation [158].

Despite this, several features of host responses to viral infection could also be affected by iron (e.g., macrophage polarization, lymphocyte proliferation, and cytokine production), potentially influencing either disease susceptibility or course [160].

In SARS-CoV-2 infection, iron deficiency seems to exaggerate the pulmonary response to hypoxic stress consequent to impaired lung function and hypoxia [161,162] and modulate cytokine production, influencing COVID-19-related inflammatory phenotypes [160]. Additionally, as reported by James et al., acute viral infection can promote an innate immune response, and iron may be withheld from the plasma through elevated hepcidin levels, leading to a functional iron deficiency and anemia of inflammation [160], with both considered adverse prognostic indicators in severe COVID-19 [163,164]. In a retrospective analysis of 259 hospitalized adults with COVID-19 in Austria, a higher risk of death (OR: 3.73; 95% CI: 1.74, 8.00) among anemic patients (specifically anemia of inflammation) compared to non-anemic ones was reported [164].

Zinc deficiency is also believed to be present among children with obesity in the same way as iron deficiency, and this has been demonstrated in studies in the pediatric age group [165]. Zinc and some zinc-dependent proteins play a role in antiviral defense and immune regulation in the respiratory tract [163]. A meta-analysis of 13 studies in China showed that pediatric patients with recurrent respiratory tract infection had low zinc levels [166]. 

Some meta-analysis and systematic review data showed that zinc could be helpful in decreasing the prevalence and incidence of pneumonia in children [154]. In adults, a decreased mortality rate in severe pneumonia was also reported [167]. A recent report by Zhang and Liu [132] suggests that zinc supplementation can ameliorate COVID-19-induced diarrhea and respiratory symptoms (i.e., cough, sore throat, and shortness of breath). In addition, the importance of zinc in the clinical course and effectiveness of drugs in adult patients with COVID-19 has been reported [168,169,170].

It should be noted that, in addition to its immunological role, zinc also participates in insulin and leptin metabolism, which can aggravate metabolic dysregulations in children affected by obesity, contributing to an inadequate inflammatory response [171].

An additional role in the immunomodulation can be also attributed to omega-3 fatty acids, which are essential lipids for humans [31]. Eicosapentaenoic (EPA) and docosahexaenoic (DHA) fatty acids are potent immunomodulators as they decrease the activity of specific nuclear transcription factors, leading to a decrease in proinflammatory molecules, such as TNF-α and IL-1β [172]. Moreover, competing with arachidonic acid (omega-6) for the metabolism of cyclooxygenase, prostaglandins and leukotrienes modulate production [173].

The immunomodulation ability depends on the omega-3/omega-6 ratio, and it was shown that a ratio of 1:15 to 1:50, typical of Western diets and found in adults [174] and children [175], has proinflammatory effects [174]. This inadequate proportion of omega-3/omega-6 impairs modulation of the immune response with a consequent risk of exacerbation of inflammatory reaction effects [31,174].

Lastly, a growing body of evidence suggests that the gut microbiota should be considered [176,177], since gut dysbiosis is common in subjects with both over and under nutrition, and diet has been shown to induce alterations of its composition [178]. Dysbiosis may contribute to the increased risk of infections affecting immune system modulation [23] so may be involved in the severity of COVID-19 infection [179,180].

## 7. Changes in Nutrition Behavior during COVID-19 and The Effect on Weight Gain

The COVID-19 pandemic indirectly affected nutritional status via several containment measures (i.e., lockdown, social isolation, school closure), contributing further to the obesogenic environment [181,182]. For this reason, several studies assessed the impact of the COVID-19 pandemic on dietary habits and lifestyle among adults, children, and adolescents [64,69,181,182] at risk of increased overweight and obesity [73,182].

Pietrobelli A et al. carried out a survey during 3 weeks of home confinement in Italy including 41 children (aged 6–18 years) with obesity [64]. It showed that intakes of potato chip, red meat, and sugar drinks increased significantly during the lockdown (*p*-value range: 0.005 to <0.001), while time spent in sports activities decreased by 2.3 (±4.6 SD) h/week (*p* = 0.003) and screen time increased by 4.8 (±2.4 SD) h/day (*p* < 0.001) [64].

At the same time, an international survey conducted in Italy, Spain, Chile, Colombia, and Brazil among 820 adolescents (aged 10 to 19 years) reported a significant increase in the consumption of fried foods and desserts (*p* < 0.001, *p* < 0.0001, respectively) during COVID-19 containment measures with lower adherence to a healthy dietary pattern [69].

Another Italian survey conducted by Di Rienzo et al. [183] during social confinement included 3533 respondents (aged 12 to 86 years). They reported an increase in junk food consumption and a lower adherence to the Mediterranean diet (MD) in the population group aged 12–17 years compared to the group aged 18–30 [183]. However, there are also findings reporting increased adherence to the MD pyramid for fruit, legumes, fish, and sweets, while cereals, nuts, and dairy intake decreased during the COVID-19 lockdown [184].

The overall findings suggested that COVID-19 led to changes in dietary habits towards an unhealthy dietary pattern and lifestyle, with individual differences probably depending on personal and familial socioeconomic status as suggested by Maffoni S. et al. [181].

During the pandemic, families have experienced the stress of increased job losses, furloughs, loss of at least one wage, and limitation of financial resources to devote to healthy food [182,183]. Moreover, the social distancing measures influenced the whole food production and distribution system by disrupting agricultural production, transportation, and sale of nutritious, fresh, and affordable foods, forcing families to rely on nutrient-poor alternatives [182,185]. Data suggest that families bought more shelf-stable less expensive, ultra-processed, and calorie-dense comfort foods [182].

On the other hand, several surveys carried out during lockdowns in Western countries have also pointed out that the negative effect of the COVID-19 pandemic may also explicate with the increasing sedentary lifestyles at pediatric age [62]. As COVID-19 spread, many countries employed restrictive policies (e.g., school closure) to slow the transmission and ease the healthcare system burden, affecting physical activity [73,186] and outdoor gatherings for leisure activities. School closure had harmful social and health consequences for children, and remote learning has been associated with weight gain, especially in overweight children [187,188], who found themselves suffering food insecurity, increased consumption of comfort food, reduced physical activity, and increased screen time both for online learning and leisure time activities, with a negative impact both on nutritional status and mental health and wellbeing [62].

Interestingly, changes in nutrition during lockdown were also described in adolescents affected by eating disorders [189]. For instance, patients with bulimia nervosa and binge eating disorder reported increased binge eating behaviors [190,191] with consequent weight gain [191].

Lastly, an interesting point, still not well addressed, is maternal nutrition, both in obese/overweight women and lean BMI pregnant women, during the COVID-19 pandemic. It has been shown that emotional eating occurred in a significant number of pregnant women during COVID-19 and was related to an excessive gestational weight gain [192], mediated by augmented intake of certain foods, such as cereals and oil, and decreases in others, such as fish and seafood [192]. Importantly, excessive gestational weight gain is considered one of the main contributors to fetal adiposity development and consequent high birth weight and increased risk of overweight and its consequences later in childhood [193].

In addition, maternal obesity has been found to be a risk factor for the increased susceptibility of pregnant women to severe COVID-19 disease [194], further highlighting the relevance of nutritional status in response to infections [194].

Overall, a qualitatively unhealthy diet, characterized by a high intake of saturated fats and refined carbohydrates including simple sugars and a low content of fiber, vitamins and minerals, and unsaturated fatty acids on the other, might expose children to nutritional inadequacies or deficiencies, thus increasing the risk of weight gain, obesity, and comorbidities, which are in turn associated with negative COVID-19 outcomes [195].

## 8. Conclusions

Obesity and COVID-19, as seen, are pandemics that negatively affect children and adolescents’ well-being [62], influencing each other in a deleterious way, both at the physiological and at the psychological level. The COVID-19 pandemic dramatically affected daily life for children and adolescents. Lockdown measures have had an impact on dietary behavior, inducing an increase in snacking and an increase in processed food consumption. Unhealthy dietary habits associated with a markable decrease in physical activity levels and an increase in sedentary behavior have been associated with weight gain, especially in overweight children. Furthermore, obesity is the most common comorbidity in severe cases of COVID-19 also in pediatrics; immune dysregulation, metabolic unbalance, inadequate nutritional status, and dysbiosis may be key factors in the interconnection between obesity and COVID-19.

Active surveillance of patients is mandatory to define the long-term impact of obesity and COVID-19. Improved access to healthy foods and nutrition counselling to support this population could be useful to prevent the negative consequences of COVID-19 on the health and lifestyle of children with obesity and to prevent the most common comorbidities associated with obesity that could worsen the clinical COVID-19 picture. Biochemical and molecular studies could be useful to create predictive models for dedicated health monitoring, preventative measures, and precision medicine for children. The introduction of telemedicine as innovative access to health care could be a prerequisite for closer patient monitoring to guarantee a systematic assessment of their health and biopsychosocial needs.

## Figures and Tables

**Table 1:** Case definition for MIS-C according to Centers for Disease Control (CDC), Royal College of Pediatrics and Child Health (RCPCH), and World Health Organization (WHO).

*Centers for Disease Control and Prevention (CDC)*	*Royal College of Pediatrics and Child Health (RCPCH)*	*World Health Organization (WHO)*
An **individual aged <21 years** presenting with **fever**, laboratory evidence of **inflammation**, and evidence of **clinically severe illness** requiring hospitalization, with **multisystem (>2) organ involvement** (cardiac, renal, respiratory, hematologic, gastrointestinal, dermatologic, or neurologic) Fever >38.0 °C for ≥ 24 h or report of subjective fever ≥ 24 h **Laboratory evidence** including at least one of the following: an elevated CRP level, ESR, fibrinogen, procalcitonin, d-dimer, ferritin, lactic acid dehydrogenase, or IL-6 levels; elevated neutrophil level; reduced lymphocyte level; and low albumin level AND **No alternative plausible diagnoses** AND **Positive for current or recent SARS-CoV-2 infection** at RT-PCR assay, serology, or antigen test; or **COVID-19 exposure within the 4 weeks** before the onset of symptoms	A **child** presenting with **persistent fever, inflammation** (neutrophilia, elevated CRP level, and lymphopenia) with evidence of **single or multiorgan dysfunction** (shock, cardiac, respiratory, renal, gastrointestinal, or neurologic disorder) with additional features This may include children fulfilling full or partial criteria for Kawasaki disease **Exclusion of any other microbial cause**, including bacterial sepsis, staphylococcal or streptococcal shock syndromes, infections associated with myocarditis such as enterovirus **SARS-CoV-2 RT-PCR testing may be positive or negative**	**Children and adolescents aged 0–19 years** with fever ≥ 3 days AND two of the following: - Rash or bilateral nonpurulent **conjunctivitis or muco-cutaneous inflammation** signs (oral, hands or feet) - **Hypotension or shock** - Features of **myocardial dysfunction**: pericarditis, valvulitis, or coronary abnormalities (including echocardiographic findings or elevated troponin/NT-proBNP) - Evidence of **coagulopathy** (PT, PTT, elevated d-dimer level) - Acute **gastrointestinal problems** (diarrhea, vomiting, or abdominal pain) AND Elevated markers of **inflammation** (as ESR, CRP, or procalcitonin) AND **No other obvious microbial cause of inflammation**, including bacterial sepsis, staphylococcal or streptococcal shock syndromes AND **Evidence of COVID-19** (RT-PCR assay, antigen test, or serology positive) **or likely contact with patients with COVID-19**

CRP = C-reactive protein, ESR = erythrocyte sedimentation rate, IL-6 = interleukin 6, NT-proBNP = *N*-terminal pro–B-type natriuretic peptide, PT = prothrombin time, PTT = partial thromboplastin time, RT-PCR = reverse transcription polymerase chain reaction.

## Data Availability

Not applicable.
